# Supplementary material for: Identification of the Most Suitable Mobile Apps to Support Dietary Approaches to Stop Hypertension (DASH) Diet Self-Management: Systematic Search of App Stores and Content Analysis
Source: Nutrients. 2023 Aug 6;15(15):3476. doi: 10.3390/nu15153476 (PMC10421018; doi:10.3390/nu15153476)
Supplement: Supplementary file 1 [file nutrients-15-03476-s001.zip › nutrients-2506225-supplementary.pdf]

Supplementary Materials, Table S1: DASH diet Apps

	App name	App store	The developer	Version date	Cost	Number of downloaded	functions: Self - monitoring	functions: DASH diet information	comments
1	My Dash Diet : Food tracker and low sodium Recipes	Android Apple	Prestige Worldwide Apps	19/04/2022	£8.48	+10,000	✓	✓	
2	Noom	Android Apple	Noom,Inc	8 Dec 2022	£0.62-£121.82	10M+	✓	✓	
3	Track – calorie Counter Nutritionnix	Android Apple	Nutritionnix	25 Oct 2021	£4.77-£23.6	---	✓	x	
4	DASH diet: weight loss plan	Android	Alebg	22/04/2022	£1.69	+10,000	✓	✓	
5	DASH Diet: Doctor Recommendation	Apple	Realized	2021	£ 10.84	--	✓	✓	
6	DASH to ten	Apple	Elencee, Inc	2020	£8.18	--	✓	✓	
7	DASH Diet Tracker	Apple	Rick Hutchinson	2021	£0.81	---	✓	x	
8	DASH Diet Recipes	Android	Riafy Technologies	2021	£1.29-52.99	+5,000	✓	✓	This app offers a variety of diets.
9	DASH diet plan and food tracker	Apple	Nikita Gnedin	2020	£7.66	---	✓	x	Nutrition information related to other diets
10	DASH daily tracker	Android	Rom Oded	18/05/2021	free	No reviews yet	✓	x	Technical issue
11	WHEELS for DASH VA	Apple	The University of Michigan	2022	--	---	✓	✓	The app is part of a research project and requires an access code.
12	WHEELS for DASH	Apple	The University of Michigan	2022	--	---	✓	✓	The app is part of a research project and requires an access code.

Supplementary Materials, Table S2: Frequency of behaviour change techniques (BCTs) (n = 19) utilised in the apps reviewed

	BCTs	Explanation	Number of Apps
1	Self-monitoring of behaviour	Ask the person to monitor and record their behaviour(s), including recording their daily food consumption, blood pressure measurements, how many cups of water they drink, and their daily number of steps.	7
2	Self-monitoring of outcome(s) of behaviour	Require the user to weigh themselves at the end of each day / week or monitor their BP reading for some weeks and record their daily weight on a graph to encourage them to eat healthily.	7
3	Problem solving	Allow the user to analyse the factors that influence [a] DASH diet adherence and create strategies (e.g. diet self-mentoring) that include overcoming barriers that prevent DASH diet adherence.	7
4	Action planning	Set a plan to cook specific recipes suitable for hypertensive patients or send notifications to complete a task(s) (record food, drink water, measure blood pressure, exercise, read articles) at a particular time.	7
5	Feedback on outcomes of behaviour	Offer feedback on the behaviour outcome, such as informing the user of how much weight they lost and/or their blood pressure reduce	7
6	Review behaviour goal(s)	Enable the user to examine their performance of agreed-upon objectives, then modify based on their achievement.	7
7	Goal setting (behaviour)	Allow the user to define goals for the behaviour to be achieved, such as setting a daily aim of eating five pieces of fruit as specified in [a] DASH diet guideline.	7
8	Review outcome goal(s)	Enable the user to assess how successfully they have adhered to DASH diet recommendations, such as how much how much weight have you lost, or how much does BP decrease, and adjust their goals accordingly.	7
9	Feedback on behaviour	Provide feedback on performance of the behaviour by displaying data in a graph (e.g., how many calories the user ate each day) or traffic lights colours to demonstrated how healthy the food was and how many calories are consumed every day.	6

	BCTs	Explanation	Number of Apps
10	Information about health consequence	Provide educational information about the benefits of adherence to the DASH diet and consequences of non-adherence to the DASH diet to control hypertension.	5
11	Prompts/cues	Send notifications when it is time to do task(s), such as eating meals at a particular time, recording food or reading articles to remind and encourage the user.	5
12	Habit formation	Repeatedly prompt the user to perform a behaviour at the same time in order to elicit a behaviour, such as eating their food at a specific time.	5
13	Instruction on how to perform a behaviour	Provide information or tips on how to follow the DASH diet and general information on how to control hypertension.	4
14	Credible source	Present information based on evidence.	4
15	Social support	Offer social support, such as providing a place to chat with a coach or their friends.	4
16	Goal setting (outcome)	Allow the user to set a goal defined by a positive outcome, such as weight loss (e.g., 0.5 kg in a week).	3
17	Reduce negative emotions	Provide suggestions for activities, such as meditation, for reducing negative feelings.	2
18	Biofeedback	Inform the user of their blood pressure reading to encourage adoption of the DASH diet.	2
19	Social incentive	Add points for the user when they answer the quiz questions or achieve their goal.	1

[a] ***DASH: Dietary Approaches to Stop Hypertension***

Supplementary Materials, Table S3: Mapping functionalities to BCTs and TDF

Functionalities	BCTs	TDF
**Self-monitoring**	Self-monitoring of behaviour<br>Self-monitoring of outcome(s) of behaviour	Behaviour regulation
**Communication with other**	Social support	beliefs about capabilities
**Goal setting**	Goal setting (outcome)<br>Goal setting (behaviour)<br>Review outcome goal(s)<br>Review behaviour goal(s)	Goals
**Food plan**	Action planning<br>Problem solving	Skills<br>beliefs about capabilities<br>Goals<br>Behaviour regulation
**Educational information**	Information about health consequences<br>Instruction on how to perform a behaviour	Knowledge
	Credible source	knowledge<br>reinforcement
**Feedback**	Feedback on behaviour<br>Feedback on outcomes of behaviour	Knowledge<br>beliefs about consequences
	Biofeedback	Skills<br>Behaviour regulation
**Reminder and Notification to log food**	Prompts / cues	Memory attention and decision process
	Habit formation	Memory attention and decision process<br>Behaviour regulation
	Action plan	goal
**Stress management**	Reduce negative emotion	Emotion

Supplementary Materials, Table S4: Data privacy and security evaluation of apps (data gathering, sharing, and security) as stated in the privacy policy.

Note: One app did not have a privacy policy available.

	Privacy and security questions	iPhone (N = 2) n	Android (N =4) n	Total (N =6) n
Availability	Is the privacy policy available without the need to download the app?			
	No	0	0	0
	Yes	2	4	6
	Is the privacy policy available within the app?			
	No	0	2	2
	Yes	2	2	4
Accessibility	Is there a short form notice (in plain English) highlighting key data practices?			
	No	0	0	0
	Yes	0	0	0
	Not applicable	2	4	6
	Is the privacy policy available in any other languages?			
	No	2	3	5
	Yes	0	1	1
Data gathering	Does the app collect personally identifiable information?			
	No	0	0	0
	Yes	2	4	6
Data sharing	Does the app share users' data with a 3rd party?			
	No	0	1	1
	Yes	2	3	5
Data security	Does the app say how the users' data security is ensured? For example, encryption, authentication, and firewall			
	No	0	2	2
	Yes	2	2	4
